# Supplementary material for: Chromatin spatial analysis by METALoci unveils sex-determining 3D regulatory hubs
Source: Nat Struct Mol Biol. 2026 Feb 24;33(4):577–89. doi: 10.1038/s41594-026-01749-z (PMC13095655; doi:10.1038/s41594-026-01749-z)
Supplement: Supplementary file 1 — Supplementary Figs. 1 and 2 and Data 1 and 2. [file 41594_2026_1749_MOESM1_ESM.pdf]

nature structural & molecular biology

Supplementary information https://doi.org/10.1038/s41594-026-01749-z

Chromatin spatial analysis by METALoci unveils sex-determining 3D regulatory hubs

In the format provided by the authors and unedited

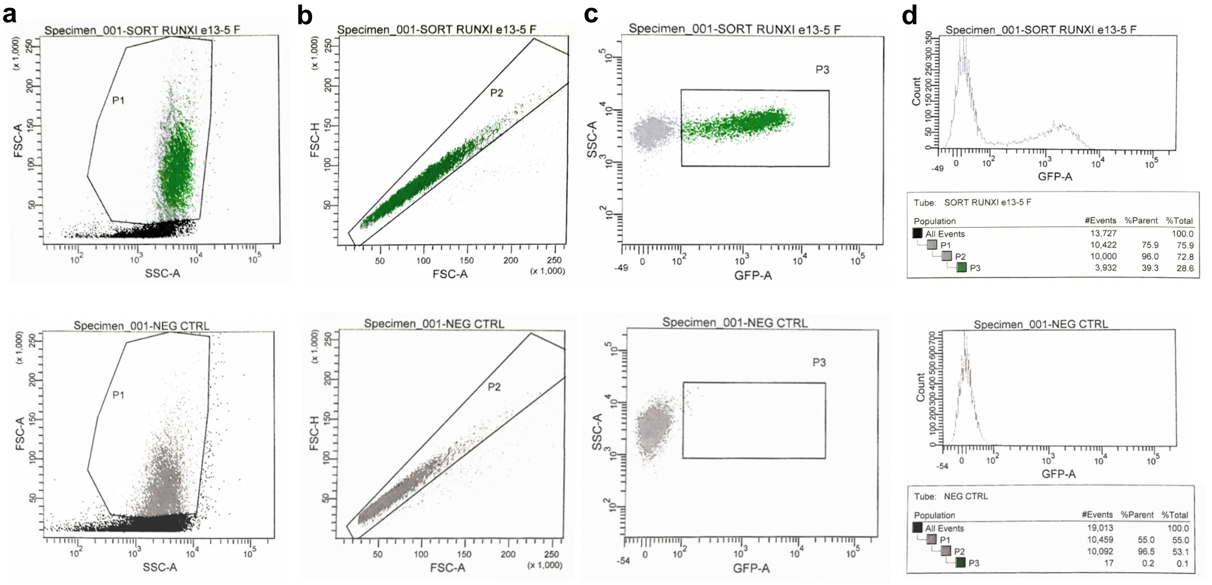

Supplementary Data Fig. 1: FACS gating strategy for XX E13.5 Runx1-GFP sample*. In each panel, the sample containing cells of interest is represented by the plot on the top row, and the negative control on the bottom row.* ***a****. Cells were gated on an FSC-A and SSC-A plot to select for cells and eliminate debris.* ***b.*** *Cells were gated on an FSC-H and FSC-A plot to select for singlets and eliminate doublets.* ***c.*** *Cells were gated on an SSC-A and GFP-A plot to identify fluorescent cells of interest.* ***d.*** *Histograms representing the cells identified by the gating strategy as event counts over fluorescence intensity (top). Representative data indicating cell population abundance for both the sample and negative control, with P1, P2, and P3 relating to the populations identified in panels a, b, and c, respectively (bottom)*

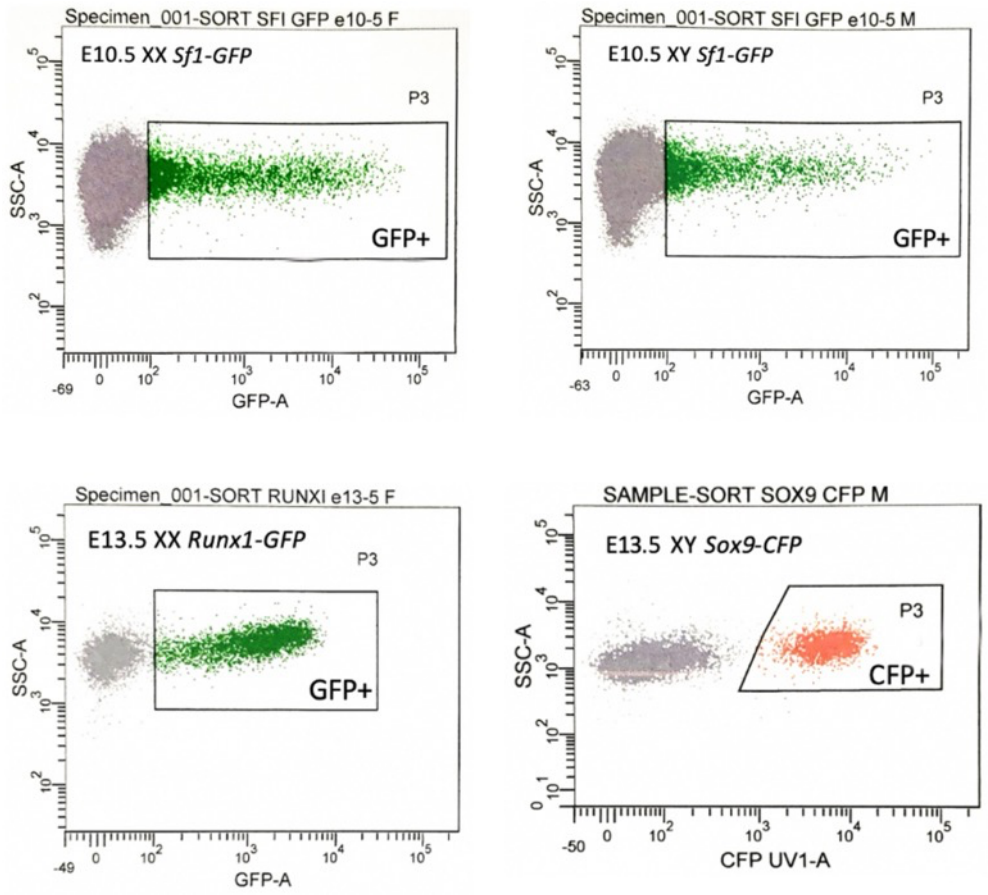

Supplementary Data Fig. 2: Representative FACS profiles for each supporting cell population collected. *Gating indicated with grey boxes. The boundaries between fluorescent and non-fluorescent populations were defined each sort based on a negative control brought with the sample. Gates were established based on the negative control, allowing fluorescent cells to be identified as outside of the negative gates.*

Supplementary Data File 1: Coordinates for HH metaloci in each sample.

The EDFile1_H3K27ac_metaloci_per_gene.zip file contains four files named:

- XX10.5_H3K27ac_HH_metaloci_per_gene.bed
- XX13.5_H3K27ac_HH_metaloci_per_gene.bed
- XY10.5_H3K27ac_HH_metaloci_per_gene.bed
- XY13.5_H3K27ac_HH_metaloci_per_gene.bed

Each BED file contains the following columns tab separated:

chr	Chromosome
start	Start coordinates
end	End coordinates
MetaLociBinNumber	Number of the bin in the METALoci layout
GeneSymbol	Gene symbol

This ZIP file can be anonymously accessed in ZENODO (10.5281/zenodo.7334816) at this URL: https://doi.org/10.5281/zenodo.7334816

Supplementary Data File 2: Coordinates for all ATAC-seq accessible peak within a metaloci and their associated gene in each sample.

The EDFile2_TF_ATAC_ML_Sites.zip file contains four files named:

- XX10.5_TF_ATAC_ML_Sites.tsv
- XX13.5_TF_ATAC_ML_Sites.tsv
- XY10.5_TF_ATAC_ML_Sites.tsv
- XY13.5_TF_ATAC_ML_Sites.tsv

Each TSV file contains the following columns tab separated:

bchr	METALoci chromosome
bstart	METALoci start coordinates
bend	METALoci end coordinates
bin	METALoci bin
pchr	ATAC site chromosome
pstart	ATAC site start coordinates
pend	ATAC site end coordinates
gene	Gene symbol

This ZIP file can be anonymously accessed in ZENODO (10.5281/zenodo.7334816) at this URL: https://doi.org/10.5281/zenodo.7334816
