# Supplementary material for: Chromatin spatial analysis by METALoci unveils sex-determining 3D regulatory hubs
Source: Nat Struct Mol Biol. 2026 Feb 24;33(4):577–89. doi: 10.1038/s41594-026-01749-z (PMC13095655; doi:10.1038/s41594-026-01749-z)
Supplement: Supplementary file 2 — Reporting Summary [file 41594_2026_1749_MOESM2_ESM.pdf]

nature portfolio

Corresponding author(s): Darío G. Lupiáñez, Marc A. Marti-Renom, Blanche Capel

Last updated by author(s): Dec 21, 2025

Reporting Summary

Nature Portfolio wishes to improve the reproducibility of the work that we publish. This form provides structure for consistency and transparency in reporting. For further information on Nature Portfolio policies, see our Editorial Policies and the Editorial Policy Checklist.

Statistics

For all statistical analyses, confirm that the following items are present in the figure legend, table legend, main text, or Methods section.

n/a	Confirmed	
☐	☒	The exact sample size (*n*) for each experimental group/condition, given as a discrete number and unit of measurement
☒	☐	A statement on whether measurements were taken from distinct samples or whether the same sample was measured repeatedly
☐	☒	The statistical test(s) used AND whether they are one- or two-sided<br>*Only common tests should be described solely by name; describe more complex techniques in the Methods section.*
☒	☐	A description of all covariates tested
☒	☐	A description of any assumptions or corrections, such as tests of normality and adjustment for multiple comparisons
☐	☒	A full description of the statistical parameters including central tendency (e.g. means) or other basic estimates (e.g. regression coefficient) AND variation (e.g. standard deviation) or associated estimates of uncertainty (e.g. confidence intervals)
☐	☒	For null hypothesis testing, the test statistic (e.g. *F*, *t*, *r*) with confidence intervals, effect sizes, degrees of freedom and *P* value noted<br>*Give P values as exact values whenever suitable.*
☒	☐	For Bayesian analysis, information on the choice of priors and Markov chain Monte Carlo settings
☒	☐	For hierarchical and complex designs, identification of the appropriate level for tests and full reporting of outcomes
☒	☐	Estimates of effect sizes (e.g. Cohen's *d*, Pearson's *r*), indicating how they were calculated

Our web collection on statistics for biologists contains articles on many of the points above.

Software and code

Policy information about availability of computer code

Data collection	No software was used for data collection
Data analysis	Most analysis were performed with standard packages for NGS analysis and/or with custom python scripts.<br><br>New code:<br>- METALoci in house developed Python 3 code available as a Jupyter notebook in GitHub (https://github.com/3DGenomes/METALoci).<br><br>The following public software was used for data analysis:<br><br>Bowtie2 version= 2.3.4.3<br>samtools versions= 1.9<br>STAR version= 2.7.10a<br>MACS2 version=2.2.7.1<br>GeoPandas (https://geopandas.org, 0.10.2)<br>NetworkX (https://networkx.org, 2.6.3)<br>libpysal (https://pysal.org, 4.6.2)<br>ESDA (https://pysal.org/esda/, 2.4.1)<br>pyBigWig library from deepTools (https://deeptools.readthedocs.io, 0.3.18).<br>flexbar version=3.4<br><br>R version=4.0.2

featureCounts version= v2.0.1
DEseq2 version= 1.28.1
ggplot2 version=3.3.6
reshape version=0.8.8
biomaRt version=2.56.3
PiGX version=0.1.1
Trim Galore! version= 0.6.5

python version= 3.7.9
fanc version=0.9.8
numpy version=1.21.6
seaborn version=0.11.2
pandas version= 1.3.5
pybedtools version=0.8.1
matplotlib version= 3.5.2
juicer version=1.5.6
deeptools version = 3.4.3
bedtools version= 2.3
SciPy version= 1.9.3

pySCENIC version= 0.12.1

For manuscripts utilizing custom algorithms or software that are central to the research but not yet described in published literature, software must be made available to editors and reviewers. We strongly encourage code deposition in a community repository (e.g. GitHub). See the Nature Portfolio guidelines for submitting code & software for further information.

Data

Policy information about availability of data

All manuscripts must include a data availability statement. This statement should provide the following information, where applicable:

- Accession codes, unique identifiers, or web links for publicly available datasets
- A description of any restrictions on data availability
- For clinical datasets or third party data, please ensure that the statement adheres to our policy

Raw data were submitted to GEO under the accession number: GSE217618 and they are available for the public

Research involving human participants, their data, or biological material

Policy information about studies with human participants or human data. See also policy information about sex, gender (identity/presentation), and sexual orientation and race, ethnicity and racism.

Reporting on sex and gender	*Use the terms sex (biological attribute) and gender (shaped by social and cultural circumstances) carefully in order to avoid confusing both terms. Indicate if findings apply to only one sex or gender; describe whether sex and gender were considered in study design; whether sex and/or gender was determined based on self-reporting or assigned and methods used. Provide in the source data disaggregated sex and gender data, where this information has been collected, and if consent has been obtained for sharing of individual-level data; provide overall numbers in this Reporting Summary. Please state if this information has not been collected. Report sex- and gender-based analyses where performed, justify reasons for lack of sex- and gender-based analysis.*
Reporting on race, ethnicity, or other socially relevant groupings	*Please specify the socially constructed or socially relevant categorization variable(s) used in your manuscript and explain why they were used. Please note that such variables should not be used as proxies for other socially constructed/relevant variables (for example, race or ethnicity should not be used as a proxy for socioeconomic status). Provide clear definitions of the relevant terms used, how they were provided (by the participants/respondents, the researchers, or third parties), and the method(s) used to classify people into the different categories (e.g. self-report, census or administrative data, social media data, etc.) Please provide details about how you controlled for confounding variables in your analyses.*
Population characteristics	*Describe the covariate-relevant population characteristics of the human research participants (e.g. age, genotypic information, past and current diagnosis and treatment categories). If you filled out the behavioural & social sciences study design questions and have nothing to add here, write "See above."*
Recruitment	*Describe how participants were recruited. Outline any potential self-selection bias or other biases that may be present and how these are likely to impact results.*
Ethics oversight	*Identify the organization(s) that approved the study protocol.*

Note that full information on the approval of the study protocol must also be provided in the manuscript.

Field-specific reporting

Please select the one below that is the best fit for your research. If you are not sure, read the appropriate sections before making your selection.

☒ Life sciences ☐ Behavioural & social sciences ☐ Ecological, evolutionary & environmental sciences

For a reference copy of the document with all sections, see nature.com/documents/nr-reporting-summary-flat.pdf

Life sciences study design

All studies must disclose on these points even when the disclosure is negative.

Sample size	Sample size were 2 biological replicates for each sample in Hi-C experiment. 2-4 technical replicates from each biological replicate were performed.<br>RNA-seq and immunofluorescence analysis of mutant gonads and WT have more than 3 biological replicates.
Data exclusions	Samples were excluded only according to the genotype assessed in control experiments.
Replication	All experiments were performed in at least two biological replicates and good reproducibility was assessed by Pearson correlation.
Randomization	Randomization was not relevant for this study because all the experiments done had to take in account the genotype and the sex of the sample, and there is no treatment involved or additional covariates expected to introduce biases in development.
Blinding	Blinding was not relevant for our study, since all comparisons were performed automatically using statistical software that is not influenced by the investigator.

Reporting for specific materials, systems and methods

We require information from authors about some types of materials, experimental systems and methods used in many studies. Here, indicate whether each material, system or method listed is relevant to your study. If you are not sure if a list item applies to your research, read the appropriate section before selecting a response.

Materials & experimental systems

n/a	Involved in the study	
☐	☒	Antibodies
☐	☒	Eukaryotic cell lines
☒	☐	Palaeontology and archaeology
☐	☒	Animals and other organisms
☒	☐	Clinical data
☒	☐	Dual use research of concern
☒	☐	Plants

Methods

n/a	Involved in the study	
☒	☐	ChIP-seq
☐	☒	Flow cytometry
☒	☐	MRI-based neuroimaging

Antibodies

Antibodies used	SOX9 (Merck millipore, AB5535, Lot : 3282152, 1:600)<br>FOXL2 (abcam, ab5096-100ug, Lot:GR270487-1, 1:150)<br>SCYP3 (abcam, ab15093, Lot: GR302438-2, 1:200)<br>Alexa Fluor 488 donkey anti goat IgG (life technologies, A11055, Lot:2134018, 1:200)<br>Alexa Fluor 555 donkey anti rabbit IgG (life technologies, A31572, Lot: 2088692, 1:200)
Validation	Primary antibodies<br>SOX9:<br>Anti-Sox9 Antibody is a well characterized affinity purified Rabbit Polyclonal Antibody that reliably detects Transcription Factor Sox-9. This highly published antibody has been validated in IHC & WB<br>FOXL2:<br>Goat polyclonal to FOXL2, host species:goat, tested applications WB, postivie control WB: K562 and HeLa cell lysates, species reactivity: Human but predicted to work on mouse, rat and cow<br>SCYP3:<br>Rabbit polyclonal to SCP3, Suitable for: IHC-P, ICC/IF,Reacts with: Mouse, Human, Synthetic peptide (Human) (C terminal), positive control: mouse testis tissue sections, IHC-P<br><br>Secondary antibodies:<br>Alexa Fluor 488 donkey anti goat IgG:<br>Applications: IHC, ICC/IF, Flow. Species reactivity:Goat, Host :Donkey IgG, Class: polyclonal, Type:Secondary antibody. Immunogen: Gamma immunoglobins heavy and light chains. Conjugate: Alexa Fluor 488

Alexa Fluor 555 donkey anti rabbit IgG:
Applications: IHC, ICC/IF, Flow. Species reactivity:Rabbit, Host :Donkey IgG, Class: polyclonal, Type:Secondary antibody. Immunogen: Gamma immunoglobins heavy and light chains. Conjugate: Alexa Fluor 555

Eukaryotic cell lines

Policy information about cell lines and Sex and Gender in Research

Cell line source(s)	G4F1 (https://doi.org/10.1073/pnas.0609277104)<br>G4F1/del306 (generated in this study)<br>G4F1/del93 (generated in this study)<br>G4F1/del104 (generated in this study)
Authentication	Cell lines were not authenticated
Mycoplasma contamination	All cells were tested for mycoplasma contamination using Mycoalert detection kit (Lonza) and Mycoalert assay control set (Lonza)
Commonly misidentified lines<br>(See ICLAC register)	No commonly misidentified cell lines were used

Animals and other research organisms

Policy information about studies involving animals; ARRIVE guidelines recommended for reporting animal research, and Sex and Gender in Research

Laboratory animals	Mus musculus CD-1, female animals from various ages for donor and embryos retransfered by tetraploid aggregation and E13.5 embryos isolated to perform experimental analysis.<br>Nr5a1-eGFP on a C57BL/6 (B6) background to FACS sort cell populations for Hi-C experiments (reference stated in methods)<br>Sox9-eCFP on a C57BL/6 (B6) background to FACS sort cell populations for Hi-C experiments (reference stated in methods)<br>Runx1-GFP also in C57BL/6 (B6) background to FACS sort cell populations for Hi-C experiments (reference stated in methods)<br>Meis1flox on a C57BL/6 (B6) background to obtain Meis1/Meis2 conditional knockout embryos (reference stated in methods)<br>Meis2flox on a C57BL/6 (B6) background to obtain Meis1/Meis2 conditional knockout embryos (reference stated in methods)<br>Stra8Cre on a C57BL/6 (B6) background to obtain Meis1/Meis2 conditional knockout embryos (reference stated in methods)<br>Zp3Cre on a C57BL/6 (B6) background to obtain Meis1/Meis2 conditional knockout embryos (reference stated in methods)
Wild animals	No wild animals were used in this study
Reporting on sex	Sex was considered for the study. The embryos from Sox9-eCFP line were males and the ones from Runx1-GFP females, since we were interested in the populations from testis and ovaries respectively.
Field-collected samples	This study did not involve field-collected samples
Ethics oversight	Mice used for tetraploid complementation assays were handled according to institutional guidelines under an experimentation licence (G0111/17 and G0051/22) approved by the Landesamt fuer Gesunheit und Soziales (Berlin, Germany), or according to the Spanish law and EU Directive 2010/63/EU, with approvals from the University Pablo de Olavide Ethics Committee and the Junta de Andalucía, with reference 09/02/2024/024.<br><br>Reporter mice used for FACS of the cell populations of the gonad were handled in accordance with National Institutes of Health guidelines and with the approval of the Duke University Medical Center Institutional Animal Care and Use Committee (A089-20-04 9N).<br><br>Mice used for the generation of the Cre/lox lines were handled in accordance with CNIC Ethics Committee, Spanish laws and the EU Directive 2010/63/EU for the use of animals in research. All mouse experiments were approved by the CNIC and Universidad Autónoma de Madrid Committees for 'Ética y Bienestar Animal' and the area of 'Protección Animal' of the Community of Madrid with reference PROEX 220/15.

Note that full information on the approval of the study protocol must also be provided in the manuscript.

Plants

Seed stocks	*Report on the source of all seed stocks or other plant material used. If applicable, state the seed stock centre and catalogue number. If plant specimens were collected from the field, describe the collection location, date and sampling procedures.*
Novel plant genotypes	*Describe the methods by which all novel plant genotypes were produced. This includes those generated by transgenic approaches, gene editing, chemical/radiation-based mutagenesis and hybridization. For transgenic lines, describe the transformation method, the number of independent lines analyzed and the generation upon which experiments were performed. For gene-edited lines, describe the editor used, the endogenous sequence targeted for editing, the targeting guide RNA sequence (if applicable) and how the editor was applied.*
Authentication	*Describe any authentication procedures for each seed stock used or novel genotype generated. Describe any experiments used to assess the effect of a mutation and, where applicable, how potential secondary effects (e.g. second site T-DNA insertions, mosiacism, off-target gene editing) were examined.*

Flow Cytometry

Plots

Confirm that:

☒ The axis labels state the marker and fluorochrome used (e.g. CD4-FITC).

☒ The axis scales are clearly visible. Include numbers along axes only for bottom left plot of group (a 'group' is an analysis of identical markers).

☐ All plots are contour plots with outliers or pseudocolor plots.

☒ A numerical value for number of cells or percentage (with statistics) is provided.

Methodology

Sample preparation	The Sf1-eGFP (Nr5a1-eGFP) and Sox9-eCFP reporter mouse lines previously generated were maintained on a C57BL/6 (B6) background64,65. The Runx1-GFP reporter mouse was generously gifted by Dr. Humphrey Yao at NIEHS20 (availability at MMRRC_010771-UCD). Timed matings were generated with reporter males and wild-type CD1 females. The morning of a vaginal plug was considered E0.5. Embryos were collected at E10.5 and E13.5, with genetic sex determined using PCR for the presence or absence of the Y-linked gene Uty. Gonads were dissected from E10.5 or E13.5 embryos, and the mesonephros was removed using syringe tips. The gonads were incubated in 500ul 0.05% trypsin for 6-10 min at 37°C, then mechanically disrupted in 1X PBS/10% FCS. The cell suspension was pipetted through a 40µm filter top and the supporting cells were collected with FACS.
Instrument	GFP-positive cells were sorted using a Becton Dickinson (BD) DiVa and analyzed with the BD Fortessa X-20. CFP-positive cells were sorted using a B-C Astrios and analyzed with the BD Fortessa X-20
Software	Data was analyzed using the BD FACSDiVa software
Cell population abundance	Post-sort purity was determined to be greater than 97% by re-analyzing the post-sort fraction by FACS. Cell population abundance was on average as follows: 2.8% for E10.5 XX, 2.5% for E10.5 XY, 30% for E13.5 XX, and 20% for E13.5 XY.
Gating strategy	Cells were gated on an FSC-A and SSC-A plot to select for cells and eliminate debris. Cells were gated on an FSC-H and FSC-A plot to select for singlets and eliminate doublets. Cells were gated on an SSC-A and GFP-A/CFP UV-A plot to identify fluorescent cells of interest. The boundaries between fluorescent and non-fluorescent populations were defined each sort based on a negative control brought with the sample. Gates were established based on the negative control, allowing fluorescent cells to be identified as outside of the negative gates.

☒ Tick this box to confirm that a figure exemplifying the gating strategy is provided in the Supplementary Information.
